# Revision of the genus *Ficiana* Ghauri and its relationship to other genera in Empoascini (Hemiptera, Cicadellidae, Typhlocybinae)

**DOI:** 10.3897/zookeys.541.6113

**Published:** 2015-12-01

**Authors:** Ye Xu, Hui-feng Suo, Dao-zheng Qin

**Affiliations:** 1 Key Laboratory of Plant Protection Resources and Pest Management of the Ministry of Education; Entomological Museum, Northwest A&F University, Yangling, Shaanxi 712100, China

**Keywords:** Auchenorrhyncha, Cicadelloidea, morphology, distribution, taxonomy

## Abstract

The empoascine genus *Ficiana* Ghauri is reviewed based on specimens from China. One new *Ficiana* species, *Ficiana aurantia* **sp. n.** is described from Guangxi in south China. An identification key to all species in this genus is provided. The morphological characters of *Ficiana* and related genera in this tribe are discussed.

## Introduction

The empoascine genus *Ficiana* was established by Ghauri (1963) based on specimens from Coimbatore (south India) (type species: *Ficiana pruthii* Ghauri, 1963). It is a small genus in Empoascini and easily identified by having a median sulcus on frons, vein CuA of hind wing unbranched, ventral pygofer appendage absent, and subgenital plates fused (Ghauri 1963). This genus is confined to the Oriental region and only one species (the type species) has been reported so far.

The generic characters of *Ficiana* need to be revised because no additional information had been added to this genus after its establishment. In this paper, a more detailed description based on specimens from China is provided. This is the first report of this genus in the Chinese fauna. In addition, a new species of *Ficiana* from Guangxi in south China is described, and the interpretation of morphology resemblance and reconsider the evolutionary relationship of this genus with related genera in the tribe Empoascini is discussed.

## Material and methods

The specimens used in this study are deposited in the Entomological Museum, Northwest A&F University, Yangling, Shaanxi, China (NWAFU). Male genitalia dissections were carried out as described by Oman (1949) and Knight (1965). Line diagrams were drawn using Olympus PM-10AD microscope. Photographs were taken with an automontage QImaging Retiga 4000R digital camera (CCD) stereozoom microscope. The body measurements are from apex of vertex to tip of forewing. Terminology follows Zhang (1990) with the following exceptions: wing venation follows Dworakowska (1993), groups of setae on the subgenital plate follow Southern (1982), and leg chaetotaxy follows Rakitov (1998).

## Taxonomy

### Genus Ficiana

Taxon classificationAnimaliaHemipteraCicadellidae

Ghauri

Ficiana Ghauri, 1963: 472.

#### Type species.

*Ficiana pruthii* Ghauri, 1963, by original designation.

#### Description.

**Figures 1–7.**
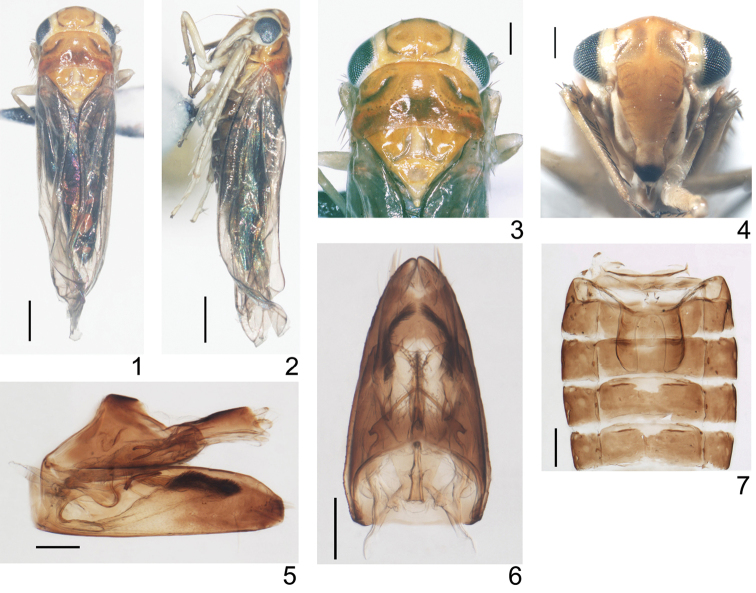
*Ficiana aurantia* sp. n. **1** male adult, dorsal view **2** male adult, left lateral view **3** head and thorax, dorsal view **4** face **5** male genitalia, left lateral view **6** male genitalia, dorsal view **7** abdominal apodemes. Scale bars: 0.5 mm (**1, 2**); 0.2 mm (**3–7**).

**Figures 8–18.**
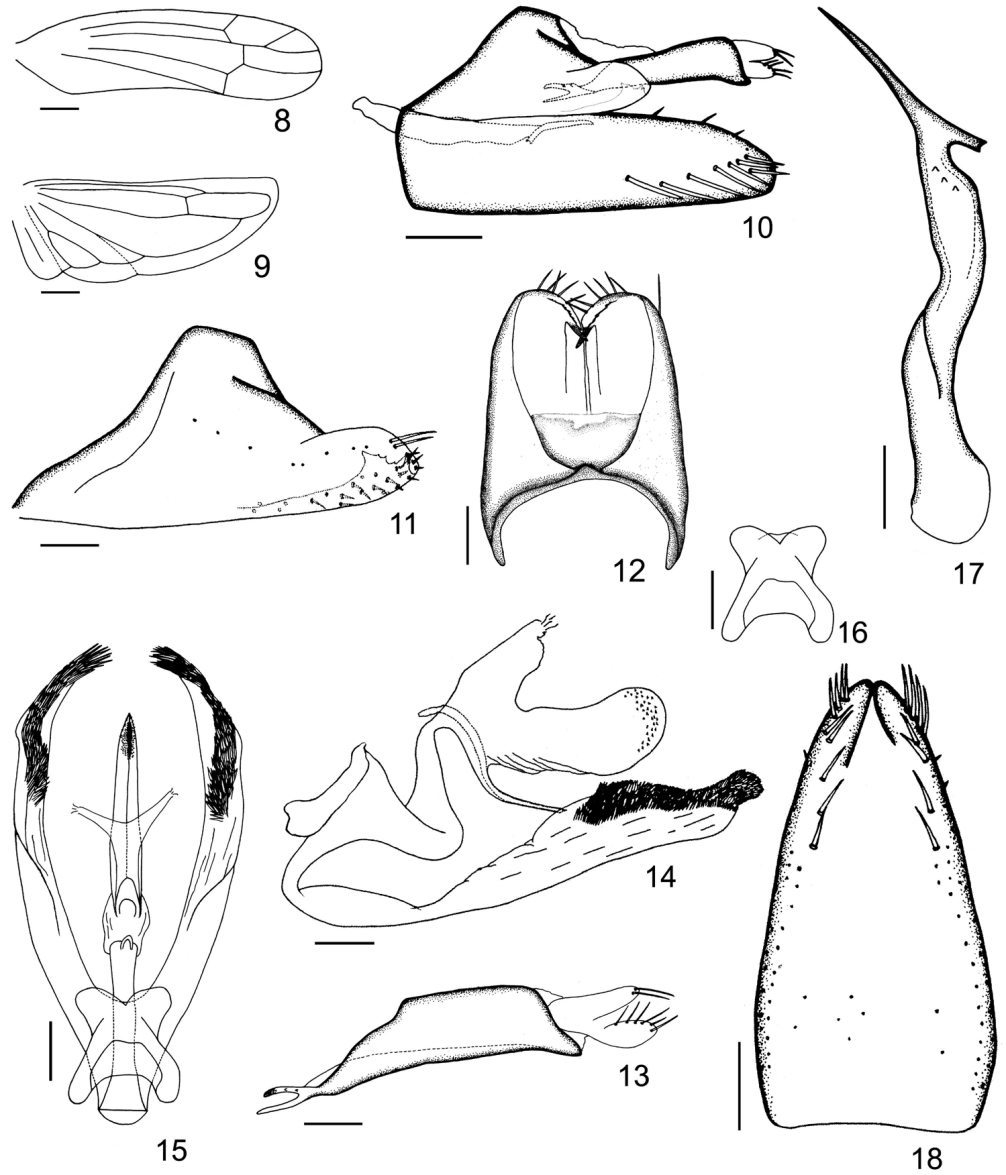
*Ficiana aurantia* sp. n. **8** forewing **9** hind wing **10** male genitalia, left lateral view **11** pygofer side, left lateral view **12** pygofer, dorsal view **13** anal tube and anal styli, left lateral view **14** aedeagus, left lateral view **15** aedeagus, dorsal view **16** connective **17** paramere **18** subgenital plates. Scale bars: 0.5 mm (**8, 9**); 0.2 mm (**10**); 0.1 mm (**11–18**).

Body robust. Head including eyes broader than maximum width of pronotum in dorsal aspect (Figs 1, 3). Crown short and broad, rounded anteriorly, anterior and posterior margins subparallel, middle length shorter than width between eyes (Figs 1, 3). Coronal suture distinct, extended onto face and terminating at level of antennal bases (Figs 1, 3, 4), transition of vertex to face rounded in profile (Fig. 2). Face broad, lateral frontal sutures convergent towards base (Fig. 4). Ocelli on margin about equidistant between eye and midline (Figs 1, 3, 4). Pronotum large with sinuate transverse depression (Figs 1–3). Scutellum with median depression. Forewing narrow, apical cells occupying nearly one-third of total length, all three apical veins arise from longitudinal m cell, veins RP, MP’ confluent for short distance pre-apically, 2nd apical cell with margins almost parallel apically (Fig. 8). Hindwing with CuA unbranched (Fig. 9). Front femur with dorsoapical pair of macrosetae, AM1 enlarged and situated on ventral margin, intercalary row with one large basal setae and eight smaller setae more distal. Hind femur macrosetae 2+1+1, row AV with 11 macrosetae near apex.

Male abdominal apodemes developed, parallel sided (Fig. 7). Pygofer short, terminally bearing rigid microsetae on each side of lobe, ventral appendage absent, dorsal bridge short (Figs 5, 6, 11, 12). Subgenital plates large and fused throughout almost in whole length except apices, A-group setae absent, B-group setae small and rigid, C-group setae sharply terminated, D-group setae not numerous (Figs 5, 6, 10, 18). Paramere short and robust, setae and sensory pits absent (Figs 5, 6, 10, 17). Connective subtrapezoidal, closely related to aedeagus (Figs 14–16). Aedeagal shaft tubular, ventro-basally produced, dorsoatrium developed (Figs 14, 15). Anal tube appendage distinct (Figs 5, 10, 13).

#### Remarks.

The original illustrations of the type species made the male genital diagnosis of this genus hard to understand, especially the configuration of the aedeagus. Based on our additional new findings, it has a median sulcus of frons (Fig. 4); all apical veins in forewing arising from longitudinal m cell, vein CuA in hind wing unbranched (Figs 8, 9); ventral pygofer appendage absent (Figs 5, 10, 11); subgenital plates large and fused throughout almost in whole length (Fig. 18); and anal tube appendage present (Figs 5, 10, 13). All of these characters ensure the new species fit the definition of *Ficiana* Ghauri (1963) and it is described here.

Ghauri (1963) noted the aedeagus was “with three pairs of elaborate appendages, as shown in the figure”. However, the figures provided by Ghauri 1963 (Figs L, K) made the characters of aedeagus rather confused. After checking the genital characters of the new *Ficiana* species (specimens deposited in NWAFU), and also Fig. L in Ghauri’s illustrations, we believe that Fig. K (aedeagus, in lateral view) was positioned upside down. Moreover, the specimens in NWAFU show the aedeagus with only two pairs of processes [one pair near middle of ventro-basal protrusion and another pair at dorso-basal prolongation of dorsoatrium (latero-distally extended to base of anal tube)] (Figs 14, 15). This paper further defines this genus by including more morphological and revised genital characters.

#### Distribution.

China (Guangxi), India.

#### Key to species of the genus *Ficiana* Ghauri (males)

**Table:** 

1	Male pygofer triangular, not truncated caudally (Figs 5, 10, 11); anal tube appendage straight, branched apically (Figs 5, 10, 13); paramere spine-like apically, subapex with a short columnar process (Fig. 17)	***Ficiana aurantia* sp. n.**
–	Male pygofer nearly hexagonal, truncated caudally (Ghauri 1963: Fig. 4E); anal tube appendage curved, unbranched apically (Ghauri 1963: Fig. 4F); paramere not spine-like apically, subapex without columnar process (Ghauri 1963: Figs 4I, J)	***Ficiana pruthii* Ghauri**

### Ficiana aurantia sp. n.

Taxon classificationAnimaliaHemipteraCicadellidae

http://zoobank.org/E8DC26D0-8EF7-44C7-A227-528A27795F46

Figs 1–7 , 8–18

#### Description.

Body length: Male 3.75–4.05 mm.

Color. General color of male orange. Crown with a brownish shallow depression beside coronal suture, sublaterally near eyes with a narrow blackish stripe on each side which is continuously extended to base of face, the stripes curved near base of vertex (Figs 1, 3, 4). Frontoclypeus with transverse linear stripes laterally, adjacent to the lateral frontoclypeal suture brown with two meniscate, brownish patches, anteclypeus black apically, lorum sordid brownish centrally (Fig. 4). Eyes dark (Figs 1–4). Ocelli circled with whitish creamy patch (Figs 1–4). Pronotum with black, sinuate transverse depression laterally, mid-posteriorly reddish-black, laterobasal angles studded with reddish patches (Figs 1, 3). Centre of scutellum with a quadrate creamy patch anteriorly (Figs 1, 3). Abdomen black (Figs 1, 2). Fore and hindwing subhyaline, vein distinct (Figs 1, 2). Legs greyish (Fig. 2).

Basal abdominal sternal apodemes reaching the end of segment 4 (Fig. 7). Male pygofer almost triangular, distal lobe bearing 2 long and approximately 20 rigid setae, caudo-ventral margin infolded, poorly sclerotized and apically bearing irregular teeth (Figs 5, 10–12); dorsal bridge occupying almost 1/4 of the lobe, caudally membranous (Fig. 12). Subgenital plates far surpassing tip of pygofer, gradually narrowing, both lateral sides curved upwards in lateral view, B-group setae (20–22) near dorsal margin of the plate, arising near base towards subapex in 2-3 rows, C-group setae (8–9) arising in apical 2/5, uniseriate in most part but biseriate near apex, D-group setae sparsely scattered in several irregular rows (Figs 5, 10, 18). Paramere broad and sinuate in most part, with 3 teeth near apex, apically strongly narrowed, long and spine-like, and a columnar process toward base (Figs 5, 10, 17). Aedeagal in lateral view, shaft tubular, curved and gradually tapering, gonopore apical; dorso-atrium laterally flattened, longer than shaft, with wrinkles and numerous tiny strumae on surface, dorso-basal prolongation bifurcated apically; baso-ventral protrusion of aedeagus longer than the shaft and doratrium in profile, sub-basally strongly curved, apical part broadened and directed dorso-caudally, widest near apex, bearing numerous, bushy setae on the dorsal side; dorsal view, ventro-basal protrusion bifurcated sub-medially, divergent and almost same width in basal 2/3, apices narrowed and curved (Figs 5, 6, 14, 15). Connective closely related to aedeagus near base on dorsal side, posterior and lateral margins concave, anterior margin incised medially (Figs 14–16). Anal tube process strongly narrowing and branched apically, dorsal branch short and tuberculate, ventral branch smooth (Figs 5, 10, 13).

#### Type material.

**Holotype.** ♂, China, Guangxi, Rongshui, 31 July, 2014, coll. Ye Xu. **Paratypes.** 4 ♂♂, same data as holotype.

#### Host plants.

Unknown.

#### Etymology.

The specific epithet is an adjective derived from the Latin word *“aurantium”*, referring to the orange body color of the new species.

#### Remarks.

This new species differs from *Ficiana pruthii* Ghauri by the male pygofer not truncated caudally (Figs 10, 11) (male pygofer truncated caudally in *Ficiana pruthii*); anal tube appendage straight and branched apically (Figs 5, 10, 13) (anal tube appendage curved, not branched apically in *Ficiana pruthii*); paramere spine-like apically, subapex with a short columnar process (Fig. 17) (paramere not spine-like apically, subapically without columnar process in *Ficiana pruthii*).

#### Distribution.

China (Guangxi).

## Discussion

Dworakowska (1970) studied the phenomenon of the fusion of the male plates in Cicadellidae. Although Dworakowska supposed this feature is “not rare in Cicadellidae, and it probably cannot say anything about the relationship among the higher taxa”. Dworakowska’s research still provided some hint for the classification of Empoascini, for the fused plates for some taxa in *Empoasca*-complex seem rather unique and distinguished them from other genera in this tribe. Seven genera, including *Ficiana* Ghauri, *Ishiharella* Dworakowska, *Dialecticopteryx* Kirkaldy, *Mahmoodia* Dworakowska, *Nimabanana* Dworakowska, *Kotwaria* Dworakowska and *Daluana* Ramakrishnan share this feature. Furthermore, all of these genera show some similarities in crown proportions (short and rounded anteriorly, anterior and posterior margins subparallel, middle length distinctly shorter than width between eyes, shared pygofer characteristics (without ventral appendage), the venation of forewing (all apical veins arising from longitudinal m cell) and hind wing (vein CuA unbranched). It is likely that these genera constitute a distinct group (*Ficiana* group) in the process of evolutionary history and are more closely related than they are to other genera in the *Empoasca*-complex of the tribe.

Ghauri (1963) suggested a resemblance with the genus *Sujitettix* Matsumura and *Kybos* Fieber; however, *Sujitettix* has been treated as a junior synonym of *Apheliona* Kirkaldy by Dworakowska (1970). The genus *Ficiana* is more similar to the six genera noted above. Among these genera, *Ficiana* seems more closely related to *Dialecticopteryx* Kirkaldy, *Nimabanana* Dworakowska, *Kotwaria* Dworakowska and *Daluana* Ramakrishnan in having a distinct coronal suture.

A key to the genera of the *Ficiana* group in the tribe as follows:

### Key to genera of *Ficiana* group (males)

**Table:** 

1	Coronal suture absent	**2**
–	Coronal suture present	**3**
2	Male pygofer without hook at its upper part; paramere well developed, neither concave nor provided with setae apically (spirally twisted or bifurcated apically)	***Ishiharella* Dworakowska**
–	Male pygofer has a well developed hook at its upper part; paramere very feebly developed and provided with setae at their concave tip	***Mahmoodia* Dworakowska**
3	Anal tube with baso-ventral processes	**4**
–	Anal tube without baso-ventral processes	**5**
4	Subgenital plates fused from base to subapex, lateral margins slightly convex basally and gradually narrowed apically	***Ficiana* Ghauri**
–	Subgenital plates fused only at their bases, lateral margins strongly convex basally and abruptly constricted near mid-length	***Dialecticopteryx* Kirkaldy**
5	Aedeagal shaft with a short ventro-basal and paired slender dorso-basal processes; subgenital plates fused only at their bases	***Nimabanana* Dworakowska**
–	Aedeagal shaft simple, without processes; subgenital plates fused in basal 2/3–4/5	**6**
6	Length of vertex, pronotum and scutellum subequal; aedeagus with preatrium; paramere with distinct preapical lobe	***Daluana* Ramakrishnan**
–	Length of vertex distinctly shorter than pronotum and scutellum; aedeagus without preatrium; paramere without preapical lobe	***Kotwaria* Dworakowska**

## Supplementary Material

XML Treatment for Ficiana

XML Treatment for Ficiana aurantia
